# Bellevue Hospital Medical College

**Published:** 1871-05

**Authors:** 


BELLEVUE HOSPITAL MEDICAL COLLEGE.—At a meeting of the Faculty of this Institution, Dr. W. T. Lusk was elected Professor of Obstetrics and Diseases of women and Infancy. Dr. E. L. Keyes was also made Lecturer on Dermatology.

